# Supplementary material for: A synthetic enzyme built from DNA flips 107 lipids per second in biological membranes
Source: Nat Commun. 2018 Jun 21;9:2426. doi: 10.1038/s41467-018-04821-5 (PMC6013447; doi:10.1038/s41467-018-04821-5)
Supplement: Supplementary file 1 — Supplementary Information [file 41467_2018_4821_MOESM1_ESM.pdf]

Supplementary Information

A synthetic enzyme built from DNA flips 10^7 lipids per second in biological membranes

Alexander Ohmann,[1†] Chen-Yu Li,[2†] Christopher Maffeo,[3] Kareem Al Nahas,[1] Kevin N. Baumann,[1] Kerstin Göpfrich,[1] Jejoong Yoo,[3] Ulrich F. Keyser,[1*] Aleksei Aksimentiev[2,3,4*]

[1]Cavendish Laboratory, University of Cambridge, JJ Thomson Avenue, Cambridge CB3 0HE, UK.

[2]Center for Biophysics and Quantitative Biology, University of Illinois at Urbana-Champaign, 1110 West Green Street, Urbana, Illinois 61801, USA.

[3]Center for the Physics of Living Cells, Department of Physics, University of Illinois at Urbana-Champaign, 1110 West Green Street, Urbana, Illinois 61801, USA.

[4]Department of Physics and Beckman Institute for Advanced Science and Technology, University of Illinois at Urbana-Champaign, 1110 West Green Street, Urbana, Illinois 61801, USA.

[†]These authors contributed equally to the work.

[*]Correspondence to: ufk20@cam.ac.uk, aksiment@illinois.edu

Supplementary Table 1. Nucleotide sequence of DNA strands forming the DNA nanostructure. For 1C DNA nanostructures the sc4 strand was replaced with sc4C in the assembly mix, for 2C additionally sc2 was replaced with sc2C.

Name	Length	Sequence
sc1	47	CCTTTCCACGAACACAGGGTTGTCCGATCCTATATTACGACTCCTTT
sc2	34	TTTGGGAAGGGGTTCGCAAGTCGCACCCTAAACG
sc3	36	TCTTATCCTGCATCGAAAGCTCAATCATGCATCTTT
sc4	23	TTTATGTTGAAGGCTCAGGATGC
st1	36	TTTATCGGACATTCAACATGGAGTCGTGGTGCGACT
st2	34	TGCGAACAGGATAAGACGTTTAGAATATAGGTTT
st3	47	TTTTTCGATGCCCCTTCCCGATGCATGAAGGGCATCCTGAGCCACCC
st4	23	TGTGTTCGTGGAATTGAGCTTTT
sc2C	35	TTTGGGAAGGGGTTCGCAAGTCGCACCCTAAACGA-CholTEG
sc4C	24	TTTATGTTGAAGGCTCAGGATGCA-CholTEG
st2F	34	TGCGAACAGGATAAGACGTTTAGAATATAGGTTT-Cy3
st4F	23	TGTGTTCGTGGAATTGAGCTTTT-Cy3

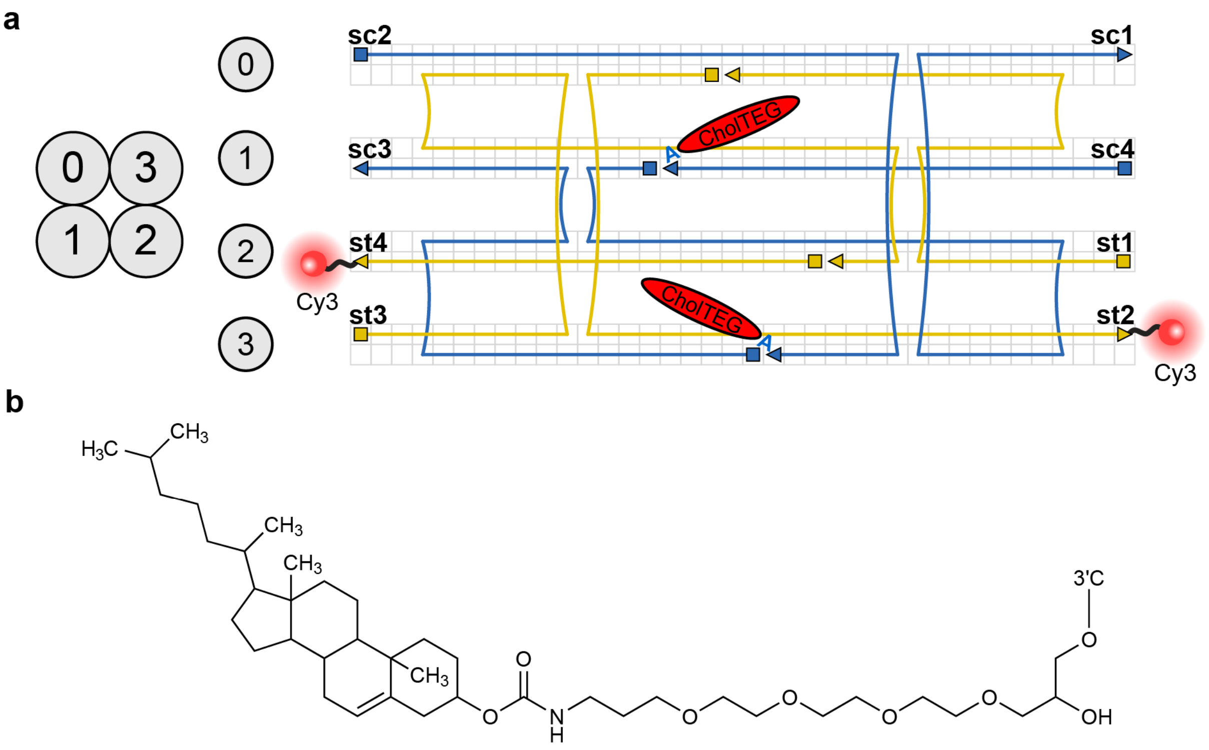

Supplementary Figure 1. Chemical architecture of DNA nanostructure. (**a**) CaDNAno design of DNA nanostructure. The nucleotide sequence of each strand is listed in Supplementary Table 1. The “CholTEG” tag indicates a cholesterol group chemically linked to a nucleotide. Cy3-tags were employed for vesicle and cell experiments. (**b**) Chemical structure of cholesterol group with TEG linker (CholTEG). The ”3′C” tag indicates the 3′ carbon of the modified nucleotide.

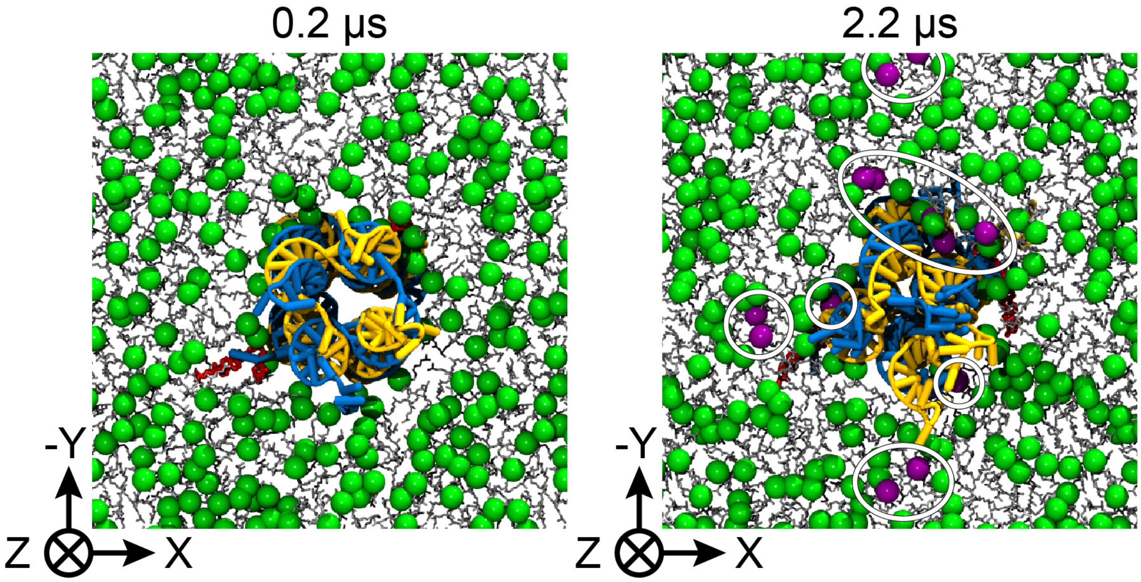

Supplementary Figure 2. Bottom-side view of the simulated system at the beginning and at the end of the free equilibration simulation. The DNA nanostructure consisting of four interconnected DNA duplexes (blue and yellow, see Supplementary Fig. 1 for structural details) is embedded in a DPhPE lipid membrane (gray) via cholesterol tags (red). The head groups of the DPhPE molecules found in the upper/lower leaflets of the bilayer at 0.2 μs are highlighted using purple/green spheres, respectively. The DPhPE head groups that resided at 0.2 μs in the upper leaflet of the bilayer are highlighted using white ellipses. The electrolyte solution is not shown for clarity.

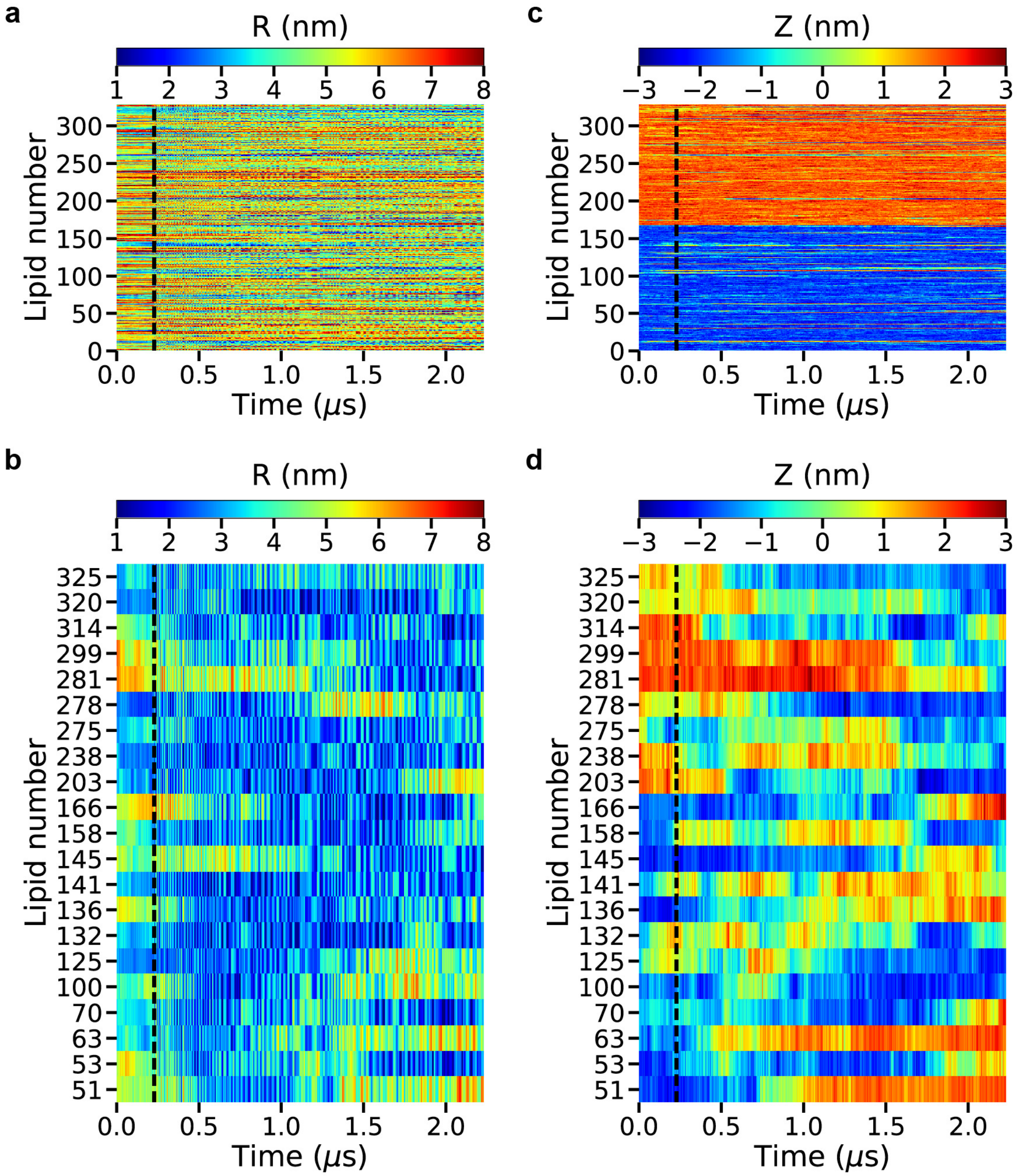

Supplementary Figure 3. Spontaneous displacement of lipids during MD simulations of the DNA nanostructure embedded in the DPhPE lipid membrane. The color maps indicate the radial distance from the center the nanostructure R (**a** and **b**) and the Z coordinate (**c** and **d**) of the lipid molecules *versus* simulation time. The radial distance was computed relative to the center of mass of the DNA nanostructure; Z = 0 was defined to be at the center of mass of the lipid membrane. Panels **a** and **c** characterize displacements of all lipids in the simulation system whereas panels **b** and **d** characterize displacements of only those lipids that moved from one leaflet of the membrane to the other (Z_{max} > 1.75 nm and Z_{min} < -1.75 nm). The black dashed lines (at 0.23 μs) indicate the beginning of free equilibration. Data shown were sampled every 0.24 ns.

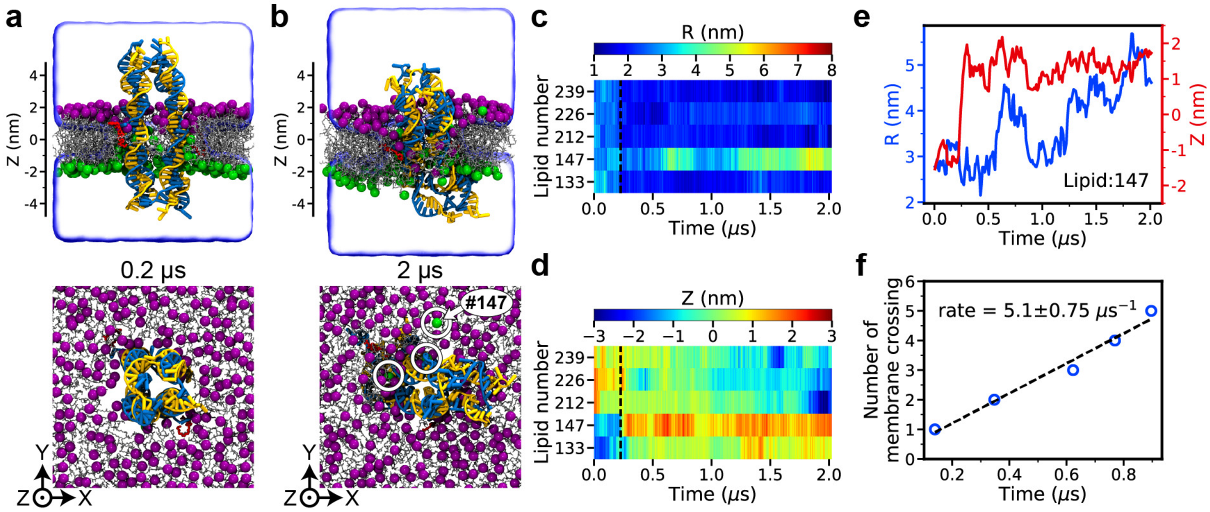

Supplementary Figure 4. Repeat all-atom simulation of lipid scrambling induced by a DNA nanostructure. (**a**,**b**) Microscopic configuration of the simulated system at the beginning (panel **a**) and the end (panel **b**) of free equilibration simulation. (Top) Cut-away view of the simulated system, showing a DNA nanostructure (blue and yellow, see Supplementary Fig. 1 for structural details) embedded in a DPhPC lipid membrane (gray) via cholesterol tags (red). The head groups of the DPhPC molecules found in the upper and lower leaflets of the bilayer at 0.2 μs are highlighted using purple and green spheres, respectively. The semitransparent molecular surface illustrates the volume occupied by 1 M KCl electrolyte, revealing a toroidal water-filled pore surrounding the nanostructure. (Bottom) Top-view of the simulated system; the electrolyte solution is not shown for clarity. The DPhPC head groups that reside at 0.2 μs in the lower leaflet of the bilayer are highlighted using white circles. (**c**,**d**) The radial distance from the center of mass of the nanostructure *R* (panel **c**) and the *Z* coordinate (panel **d**) of the phosphorus atoms of several lipids during the simulation. The black dashed lines (at 0.22 μs) indicate the beginning of free equilibration. The lipids featured in the plot moved from one leaflet to the other (Z_{max} > 1.75 nm and Z_{min} < -1.75 nm). Data shown were sampled every 0.24 ns. (**e**) *R* and *Z* traces of one lipid molecule (number 147, see panel **b**) undergoing a complete passage from the lower to the upper leaflets of the bilayer. The data shown was sampled at 0.24 ns and averaged in 9.6 ns blocks. (**f**) The cumulative number of inter-leaflet lipid transfer events *versus* simulation time. The black line shows a linear fit. The simulated transfer rate is 5.1 lipids per μs. A lower inter-leaflet transfer rate (in comparison to the DPhPE system) could result from the overestimation of the attractive interactions between DNA phosphates and the head groups of DPhPC lipids.

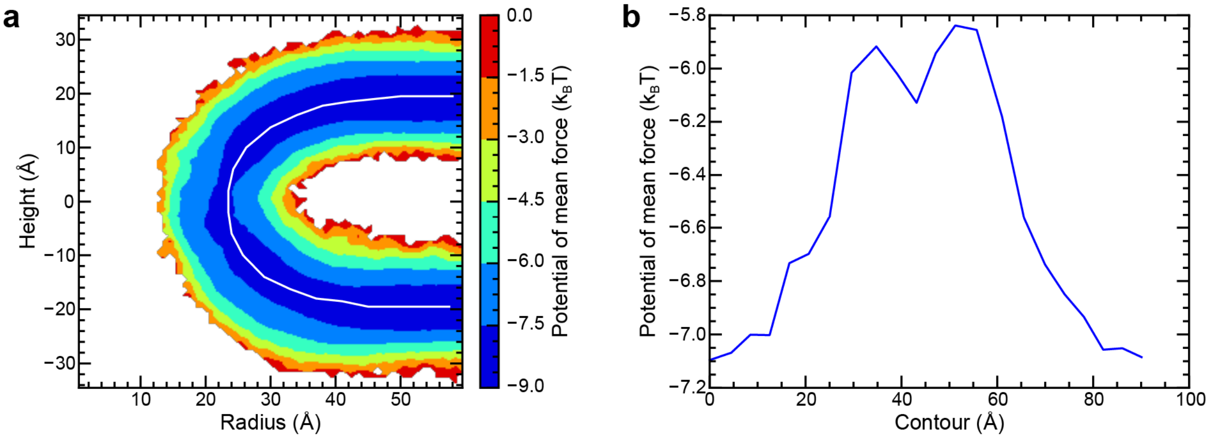

Supplementary Figure 5. Energetics of lipid scrambling. (**a**) Two-dimensional potential of mean force (PMF) for a lipid headgroup near the toroidal pore. The PMF was obtained by Boltzmann inversion of the average density of lipid phosphorus atoms. To obtain the density, the system was translated at each frame of the trajectory so that the center of mass of all lipid atoms within 2 nm of DNA was at the origin. Following that, a two-dimensional histogram was constructed by binning the radial- and *Z*-coordinates of each phosphorus atom during the 2-μs MD trajectory. An approximate transition path for moving a lipid molecule from one leaflet to the other is drawn as a white line. The transition path was obtained by selecting all bins with a density greater that 1% of the maximum and iteratively removing bins from the edges until a single path of bins remained. (**b**) Free energy profile along the transition path for a lipid moving from one leaflet to the other. To compute the free energy profile, all bins in the 2D histogram were assigned to the nearest transition path bin. The free energy at each transition path bin was calculated from the Boltzmann-weighted average of the free energies of its assigned bins from panel **a**. The contour at zero corresponds to the bin with radial and *Z*-coordinates of 59 and -20 Å, respectively.

Supplementary Note 1. All atom MD simulations of lipid scrambling.

The caDNAno design of the DNA nanostructure (Supplementary Fig. 1a) was converted to an idealized all-atom representation using a previously described method[1]. To describe the cholesterol groups covalently attached to DNA, chemical models of the attachments, including all atoms of the linkers, were created; force field parameters were obtained using the CHARMM General Force Field (CGenFF) webserver[2]. Following the design of the DNA nanostructure, cholesterol groups were added to termini of select DNA strands; the cholesterol groups were initially placed extended in opposite directions away from the DNA nanostructure.

Before inserting into a lipid membrane, the all-atom model of the DNA nanostructure was simulated for 1 ns in vacuum using the ENRG MD method[3], which allowed the structure to globally relax its conformation. The DPhPC and DPhPE lipid membranes were prepared by replicating a small patch of a pre-equilibrated lipid bilayer. After merging the DNA nanostructure with the lipid membranes, lipid molecules located either within 3 Å of the nanostructure or inside the nanostructure were removed. For the DPhPC membrane system, Mg^{2+}-hexahydrates[4] were placed at random positions near the channels in the amount required to exactly compensate its electrical charge; the DPhPE membrane system contained no magnesium ions. Following that, water and 1 M KCl were added to both DPhPC and DPhPE systems using the Solvate and Autoionize plugins of VMD.

Upon assembly, the systems were minimized using the conjugate gradient method for 1200 steps to remove steric clashes. During the minimization process, all non-hydrogen atoms of the DNA nanostructure were harmonically restrained (with spring constant k_{spring}= 1 kcal mol^{-1} Å^{-2}) to their initial coordinates. After minimization, the systems were equilibrated in the constant number of atoms, pressure (P = 1 atms) and temperature (T = 295 K) ensemble. The pressure and temperature were maintained using the Nosé-Hoover Langevin piston[5,6] and Langevin thermostat[7], respectively. The ratio of each system's dimensions was kept constant within the plane of the membrane (x-y plane); the system's dimension normal to the membrane (Z axis) was not constrained. Initially, the systems were equilibrated for 205 ns having all non-hydrogen atoms of the DNA nanostructure harmonically restrained (k_{spring} = 1 kcal mol^{-1} Å^{-2}) to their initial coordinates, which allowed the lipid and water to adopt equilibrium configurations. Following that, the spring constants of the restraints were decreased to 0.5 and then to 0.1 kcal mol^{-1} Å^{-2}; the systems were equilibrated at each spring constant value for 4.8 ns. Next, spatial restraints were replaced by a network of harmonic restraints that maintained distances between atomic pairs at their initial values; such elastic restraints excluded hydrogen atoms, phosphate groups, atoms in the same nucleotide and pairs separated by more than 8 Å. The systems were simulated under such elastic restraints for 14.4 ns; the spring constants of the restraints were decreased from 0.5 to 0.1 and then to 0.01 kcal mol^{-1} Å^{-2} in 4.8 ns steps.

All equilibration simulations were performed using the program NAMD2[8], periodic boundary conditions, the CHARMM36 parameter set for water, ions and nucleic acids[9], CHARMM parameters for the lipid bilayer, custom parameterization of ion-DNA, ion-ion and DNA–lipid interactions[4,10]. All equilibration simulations employed a 2–2–6 fs multiple time-stepping, SETTLE algorithm to keep water molecules rigid[11], RATTLE algorithm to keep all other covalent bonds involving hydrogen atoms rigid[12], a 8–10–12 Å cutoff for van der Waals and short-range electrostatic forces. Long-range electrostatic interactions were computed using the particle mesh Ewald (PME) method[13] over a 1.2-Å resolution grid[14]. The system's coordinates were recorded every 2.4 ps.

Production simulations of the DPhPE system were performed on the Anton 2 supercomputer[15] using simulation parameters equivalent to those described above for NAMD, except that temperature and pressure were maintained using the Nosé-Hoover thermostat[16,17] and the Martyna-Tobias-Klein barostat[5]. The system's coordinates were recorded every 240 ps. Production simulations of the DPhPC system were performed on Blue Waters petascale system (UIUC) using NAMD2[8].

Supplementary Note 2. Brownian dynamics simulations of lipid scrambling.

All Brownian dynamics (BD) simulations were performed using our in-house GPU-accelerated software package Atomic-Resolution Brownian Dynamics (ARBD)[18]. The head groups of lipid molecules were represented by beads that interacted with one another via a short-range repulsive potential (Supplementary Fig. 6); a cutoff of 10 Å was used to evaluate all bead-bead interactions. All other components of the system were taken into account implicitly. A 3D grid-based potential confined diffusion of the lipid head groups to the volume explored in the corresponding all-atom MD simulations. The diffusive motion of the beads was defined by a local diffusivity map that also derived from the all-atom simulations.

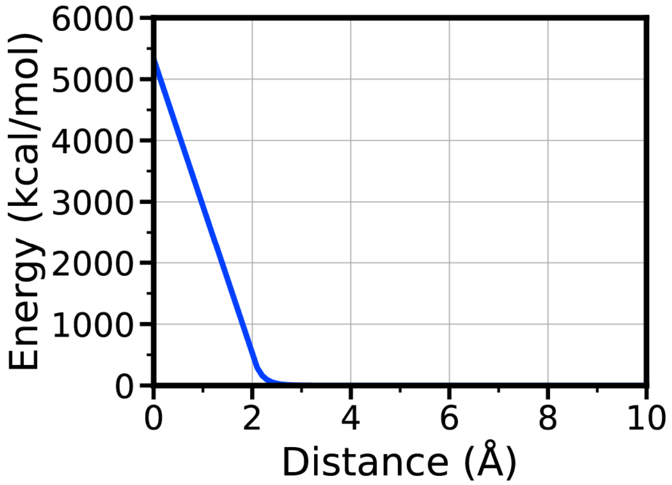

Supplementary Figure 6. Potential energy of two BD beads as a function of inter-bead distance.

At each time step of a BD simulation, the force on each bead was determined from the system configuration; the bead's coordinates were updated according to the following expression:

$$\mathbf{r}_i(t+\delta t) = \mathbf{r}_i(t) + \frac{D(r_i)\delta t}{k_B T}\mathbf{F}_i + \nabla D(r_i)\delta t + \sqrt{2D(r_i)\delta t}\,\mathbf{w}$$

where $\mathbf{r}(t)$ denotes the position at time t, $D(r)$ is the position-dependent diffusivity, $\mathbf{F}$ is the deterministic force, δt is the timestep, $k_B T$ is the thermal energy, $\mathbf{w}$ is a 3D vector with elements selected randomly from a standard normal distribution, and the subscript i indicates terms corresponding to ith bead. The deterministic force $\mathbf{F}$ had two components: one describing repulsive interaction from all other beads within the cutoff radius (Supplementary Fig. 6), and the other derived from a grid-based potential describing confinement of the toroidal pore (Supplementary Fig. 8).

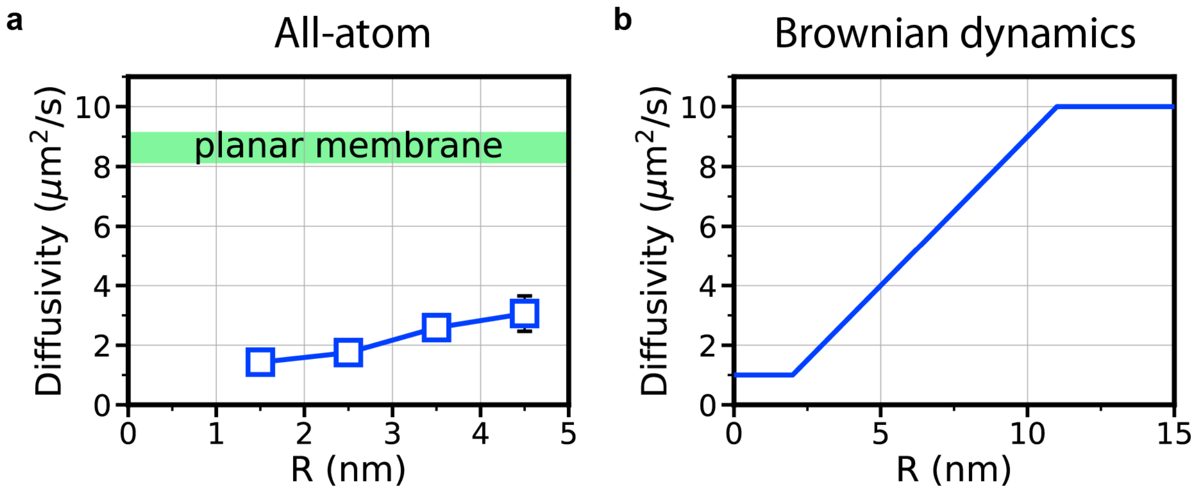

Supplementary Figure 7. Local diffusivity of lipid head groups as a function of the distance to the central axis of the DNA nanostructure *R* derived from the analysis of the all-atom MD simulation (**a**) and defined in BD simulations of the toroidal pore (**b**). In panel **a**, the error bars indicate standard deviation evaluated by bootstrapping the MD trajectory into four equal-length parts. The green area indicates the range of lipid diffusivity values observed in the simulations of a planar DPhPC bilayer system. The membrane patch simulated using the all-atom MD method is too small for the lipids to attain diffusive properties of a free bilayer system. Calculations of local diffusivities are described in Supplementary Note 3.

In all BD simulations of the toroidal pore system, the simulation time step was 200 fs; the beads' coordinates were recorded every 2.4 ns. Position-dependent diffusivity $D(r)$ was assumed to depend only on the distance from the central axis of the pore. The specific functional form, Supplementary Fig. 7b, was derived from the analysis of the all-atom MD simulations of the toroidal pore, Supplementary Fig. 7a, and from the simulations of the planar lipid membranes, Supplementary Fig. 10a. In the BD simulations of a planar lipid membrane, Supplementary Fig. 10b, the timestep was 2 fs; beads coordinates were recorded every 2.4 ps.

Generation of the 3D grid-based potential. The 3D grid-based potential was generated using the gridData module of Python. The following procedures describe generation of the 3D potential for the L = 12 nm system. All other systems were generated following the same steps but for different numerical values of L.

Step 1: To generate a grid-based potential in the 12 nm × 12 nm × 10 nm volume, we first generated a potential grid for a slightly larger volume: (12 + 2 × padding) × (12 + 2 × padding) × (10 + 2 × padding) nm^3, where padding of 1 nm was used as a buffer zone to avoid numerical errors at the boundaries of the grid. The volume was then discretized with a resolution of 0.1 nm to span Cartesian coordinates from the following range: -7 nm $\leq X < 7$ nm, -7 nm $\leq Y < 7$ nm and -6 nm $\leq Z < 6$ nm, using the gridData module. Each grid point was assigned a value of the potential energy according to its coordinate. In order to mimic the bilayer membrane, we assigned grid points satisfying the following condition: -2.2 nm $< Z < -1.8$ nm or 1.8 nm $< Z <$ 2.2 nm, a value of 100 kcal mol^{-1} and zero to all other grid points (Supplementary Fig. 8a).

Step 2: The inner surface of a toroidal pore was generated using the following catenoid function: $R - c \times \cosh(Z/c)$, where $R = (X^2 + Y^2)^{0.5}$ and constant c = 2. All grid points satisfying the following conditions: 2.25 nm < $R - c \times \cosh(Z/c)$ < 2.75 nm and −1.8 nm < Z < 1.8 nm, were set to 100 kcal mol^{-1}, producing a potential grid shown in Supplementary Fig. 8b.

Step 3: All grid points satisfying $R - c \times \cosh(Z/c)$ < 2.25 nm were set to 0, producing a toroidal pore (Supplementary Fig. 8c).

Step 4: To enlarge the volume accessible to head group beads, the grid potential was convoluted with a spherical function of 0.9 nm radius using the scipy.signal.fftconvolve function of Python; the resulting potential grid is shown in Supplementary Fig. 8d.

Step 5: To reverse the potential energy difference, all grid points that had values larger than 1 kcal mol^{-1} were set to 0 and all grid points that had values less than 1 kcal mol^{-1} were set 100 kcal mol^{-1}. Following that, the grid was convoluted with the following Gaussian window: $\exp(-0.5*(\mu/\sigma)^2)$, where μ = 1.1 nm and σ = 0.2 nm, to smooth the boundary between the regions of low and high energy (Supplementary Fig. 8e).

Step 6: To remove the numerical errors produced by the convolution procedures, the grid was trimmed to target dimensions by removing the padding which had been added initially. The final potential grid is shown in Supplementary Fig. 8f.

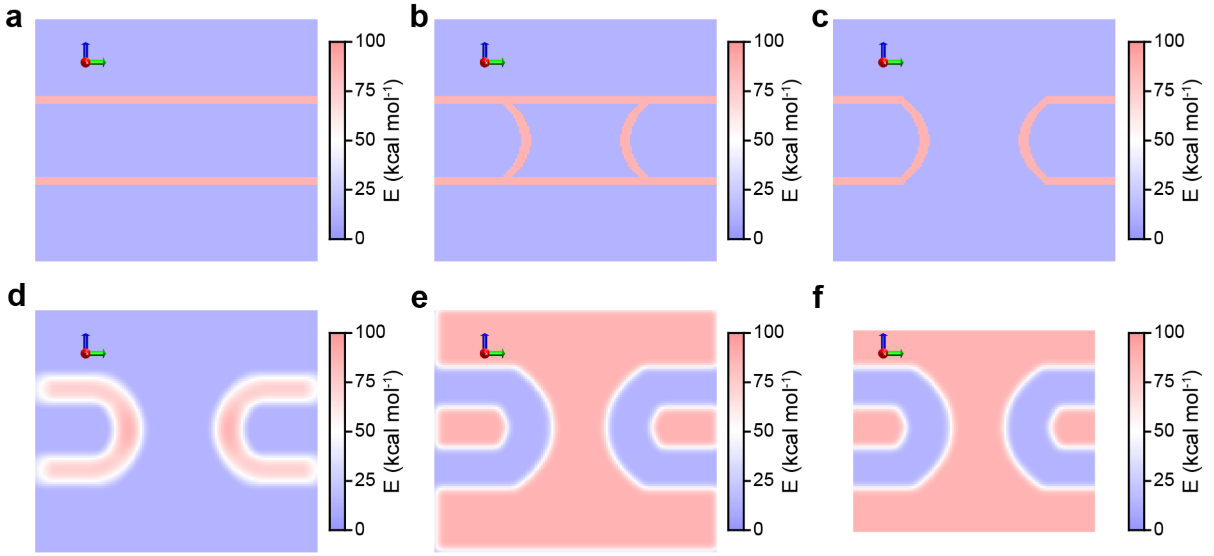

Supplementary Figure 8. Generation of a 3D grid-based potential for BD simulation of the toroidal pore. The sequence of images illustrates the six steps of the potential generation procedure. Each image shows the same cross section of the 3D potential map; the plane of the cross section passes normal to the membrane through the central axis of the toroidal pore.

Initialization of the BD simulation. For the planar lipid system (illustrated in Supplementary Fig. 10b), the initial coordinates of each beads were taken from the all-atom DPhPC system (Supplementary Fig. 10a). For the toroidal pore systems, the lipid patch size and the initial coordinates of the beads in the L = 12 nm system were taken from the all-atom system (Fig. 2 in the main text). Other systems (L = 16, 20, 24 and 36 nm) were made by increasing the lipid patch size and the number of the beads, see Supplementary Table 2.

Supplementary Table 2. Systems used for BD simulations of lipid scrambling.

L (nm)	Area (nm^2) per pore	Pore-to-lipid ratio, r
12	144	0.0030
16	256	0.0017
20	400	0.0011
24	576	0.0008
36	1296	0.0003

The initial X and Y coordinates of each bead in the BD systems were randomly generated from a homogeneous distribution within the entire range of the system dimension L. The initial Z coordinates were set to be either 2 nm or −2 nm with equal probability. Although the initial number of beads in each leaflet could differ slightly between the leaflets, the beads attained equilibrium partitioning within the first several nanoseconds of the BD simulation.

Validation of the BD approach. The BD model of the toroidal pore was validated by comparing the equilibrium local concentration of the BD beads to the concentration of the phosphorus atoms of the lipid head groups observed in the all-atom MD simulation. Supplementary Figure 8a shows the local concentration of the phosphorus atoms observed in the ~2 μs unconstrained equilibration of the DPhPE system (main text Fig. 2) sampled one frame per 0.24 ns. Supplementary Figure 8b shows the local concentration of the BD beads within the last 48 μs of the BD simulation of the L = 12 nm system (main text Fig. 3b) sampled one frame per 2.4 ns. For details of the calculation of the local concentrations, see Supplementary Note 3.

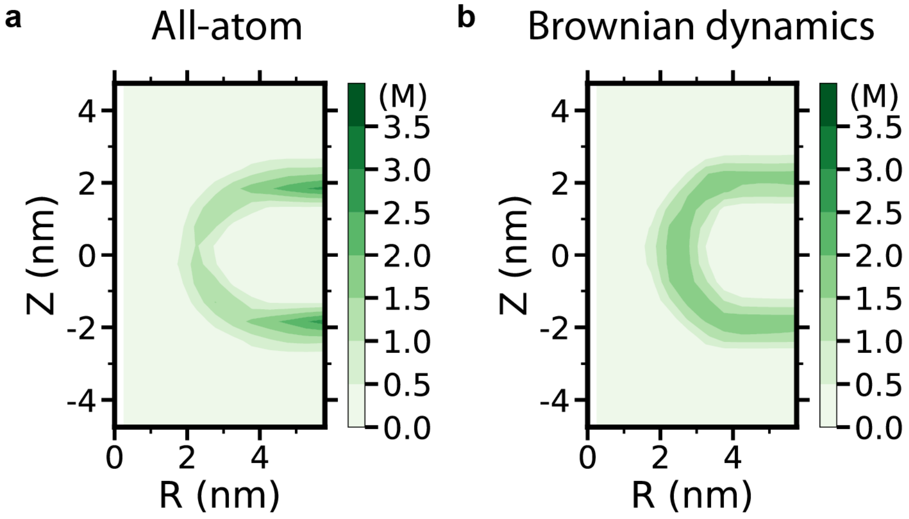

Supplementary Figure 9. Steady-state local concentration of phosphorus atoms of the lipid head groups in all-atom (**a**) and Brownian dynamics (**b**) simulations of the L = 12 nm system. The data shown were radially averaged (see Supplementary Note 3 for details).

The timescale of the BD simulation was validated by comparing the 2D diffusivity of lipids in a planar lipid bilayer system to the result of the all-atom MD simulation. For the all-atom MD simulation, the DPhPC lipid bilayer membrane was solvated in 50 mM $MgCl_2$ and 1 M KCl

solution. The final system measured 10 nm × 10 nm × 10 nm and contained ~100,000 atoms (Supplementary Fig. 10). Under periodic boundary condition, the lipid membrane was periodic in X and Y directions. Upon assembly, the system was minimized using the conjugate gradient method for 2400 steps to remove steric clashes. Then, the system was simulated in the NPT ensemble without any constraint as described in the main text Method section for 137 ns. The trajectory of the last 100 ns was used to calculate the mean squared displacement (MSD) and the diffusivity (Supplementary Fig. 10c,d). The BD system was built by taking the coordinates of the phosphorus atoms from the all-atom simulation, Supplementary Fig. 10b, as described previously. The position-dependent potential was also made by the procedure described before, skipping the steps required for generation of the toroidal pore (Steps 2 and 3). The BD system was simulated for 190 ns with a 2 fs timestep; the coordinates of the beads were recorded every 2.4 ps. Similar to the analysis of the all-atom MD trajectory, the last 100 ns of the BD trajectory were used to calculate the mean squared displacement (MSD) and the diffusivity, Supplementary Fig. 10c and d. The resulting lipid diffusivities obtained from the BD and all-atom MD simulations are in good quantitative agreement with one another.

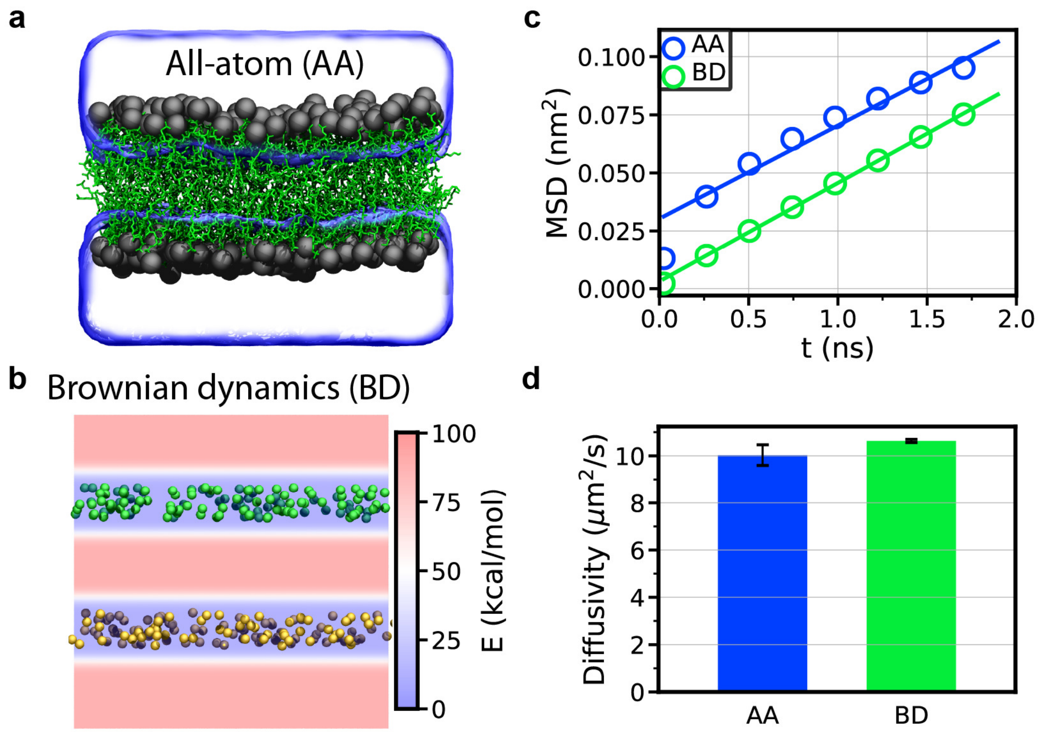

Supplementary Figure 10. Time scale comparison of the all-atom MD and BD approaches. (**a**) All-atom model of a planar DPhPC lipid bilayer. The head and tail parts of the lipids are colored in black and green, respectively. The electrolyte solution (1 M KCl and 50 mM $MgCl_2$) is shown using a semitransparent molecular surface, ions are not explicitly shown. (**b**) BD model of a planar lipid bilayer system. The green and yellow spheres represent the phosphorus atoms of the lipid bilayer membrane in the upper and lower leaflet, respectively. The color map illustrates a cross section of the potential energy grid that mimics the presence of the water-lipid interface. (**c**) Mean squared displacement (MSD) of the phosphorus atoms (AA) and BD beads (BD) versus time. (**d**) The 2D diffusivity of lipid head groups in all-atom (AA) and BD simulations. The error bars represent the standard deviation of the diffusivity values among the four quarters of the original trajectories.

Supplementary Note 3. Analysis of simulation trajectories.

Calculations of lipid diffusivity. The local diffusivity of lipids was calculated following a previously described protocol[19,20]. In the case of unrestrained Brownian motion, the diffusivity can be calculated from the Einstein relation:

$$2d_f D = \lim_{t\to\infty} \frac{1}{t} \langle \left(r(r) - r(0)\right)^2 \rangle$$

where d_f is the number of translational degrees of freedom and $r(t)$ is the position of the molecule at time t. In our simulations, however, the phosphorus atoms of the lipid are not free to move in all three dimensions as their motion is confined to the surface of the lipid bilayer. Nevertheless, we can use the above expression to obtain an approximate dependence of the lipid diffusion constant on the radial distance from the center of the nanopore and to compare lipid diffusion in the all-atom and BD systems.

To determine position-dependent diffusivity from an all-atom MD trajectory, all frames of the trajectory were first aligned so that the center of the DNA nanostructure (or of the lipid membrane in the systems that did not contain the nanostructure) was located at the origin. The BD trajectories did not require alignment. Then, trajectories of individual phosphorus atoms of the lipid molecule or BD beads were extracted; the trajectories were then divided into 20 ns segments. Each segment was categorized to belong to one of the radial bins based on the average radial distance of the atom or the bead; 1 nm bin spacing was used. Position-dependent characterization was not carried out in the case of the planar lipid membranes (Supplementary Fig. 10). Following that, the mean square displacements MSD was calculated for each segment and averaged over all the N molecules in each bin as[21]

$$\mathrm{MSD}(t) = \frac{1}{N}\frac{\Delta t}{T-t}\sum_{i=1}^{N}\sum_{t_0=0}^{T-t-1} |r(t_0) - r(t_0+t)|^2$$

The first sum runs over the N molecules and the second sum runs over all time frames smaller than $T - t$, where T is the sampling time (20 ns), t_0 is the time of the first frame in the trajectory segment and Δt is the time interval between the consecutive frames of a simulation trajectory. The slope of a linear least-squares fit to the MSD dependence on time was used as a measure of the effective diffusion coefficient for each radial bin: the 3D diffusivity was 1/6 of the slope. For the planar lipid membrane systems (Supplementary Fig. 10) only X and Y coordinates were taken into account during the analysis, corresponding to diffusion in two dimensions. In that case, the diffusivity was 1/4 of the slope.

Calculation of local concentration. The local concentration was computed as described previously[22]. We divided the simulation system into a collection of 5 Å × 5 Å × 5 Å volumes and calculated the average concentration of the particles in each volume using all available frames of the simulation trajectory. We averaged the 3D concentration in the cylindrical coordinate system over the azimuthal angle to obtain the mean concentration within the R-Z plane, as described previously[23]. Finally, we used the contouf function from the python matplotlib package to generate the local concentration plots, which were then used to display the data.

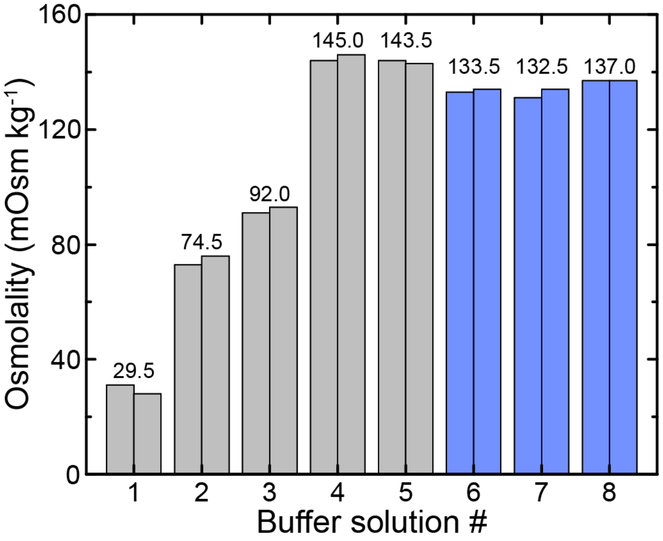

Supplementary Figure 11. Osmolality measurements of buffer solutions used in dithionite reduction assays. A freezing point osmometer (Automatic Micro-Osmometer Type 11, Roebling) was calibrated at 0 mOsm kg^{-1} with distilled water and at 300 mOsm kg^{-1} with a standard solution. Columns represent two measurement repeats and values their mean (for buffer conditions see Supplementary Table 3). Blue bars denote solutions that giant unilamellar vesicles were in contact with.

Supplementary Table 3. Buffer solutions used in dithionite reduction assays.

Buffer #	Name	Buffer conditions
1	HEPES buffer	20 mM HEPES pH 7.4
2	TE20	20 mM $MgCl_2$<br>10 mM Tris pH 8.0<br>1 mM EDTA
3	Dithionite dilution solution	50 mM glucose<br>20 mM HEPES pH 7.4<br>4 mM $MgCl_2$
4	Dithionite solution	50 mM glucose<br>20 mM HEPES pH 7.4<br>4 mM $MgCl_2$<br>15 mM dithionite<br>15 mM Tris pH 10.0
5	Glucose solution	100 mM glucose<br>20 mM HEPES pH 7.4<br>4 mM $MgCl_2$
6	Sucrose solution	100 mM sucrose<br>20 mM HEPES pH 7.4
7	Incubation solution	7 µl TE20 (#2)<br>1 µl 0.5 % OPOE in TE20<br>42 µl glucose solution (#5)
8	Measurement solution	20 µl sucrose solution (#6)<br>50 µl incubation solution (#7)<br>30 µl dithionite solution (#4)

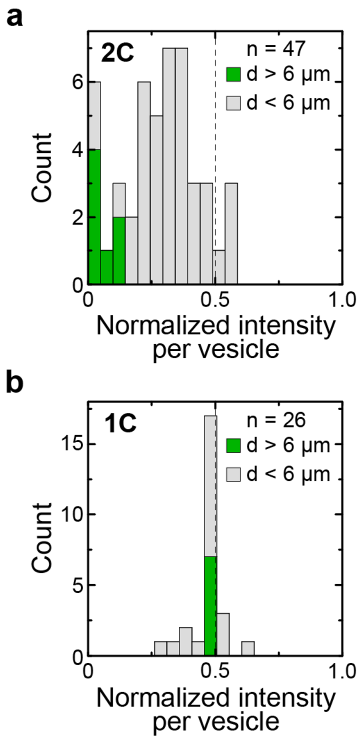

Supplementary Figure 12. Histograms of residual, normalized NBD fluorescence intensity at 35 minutes after dithionite addition for vesicles incubated with the 2C (**a**) or the 1C (**b**) design. Data for larger vesicles (diameter d > 6 µm, green) are the same as presented in main text Fig. 4d. Additionally, values for smaller vesicles (d < 6 µm, gray) are shown. Data were acquired from the same experiments as those described in the caption to main text Fig. 4d. The smallest vesicle included in the analysis was 2.6 µm in diameter. Almost all vesicles incubated with the 1C design showed a fluorescence intensity reduction to the 0.5 value whereas the majority of vesicles incubated with the 2C design displayed a reduction considerably below the 0.5 value. Bin size is 0.049.

Supplementary Note 4. Alternative dithionite reduction protocol.

In the alternative experimental protocol, the final concentration of dithionite was the same, however, the amount of dithionite solution added was greater (see Supplementary Table 4). The greater amount of added solution caused the vesicles to move upon dithionite addition in most cases. Therefore, it was rarely possible to compare the intensity before and after addition for one and the same vesicle. Alternatively, *z*-stacks of several areas containing vesicles were acquired before and one hour after dithionite addition. NBD fluorescence intensities before and after dithionite addition were determined similarly as described in the main text. Intensity values were only background subtracted but not normalized (Supplementary Fig. 13a). The fairly large spread in the intensity distribution can be explained by the use of a 20x air objective (UPLSAPO20X, NA = 0.75) in these experiments which resulted in a larger focal volume in z-direction. This caused the fluorescence intensity to depend on the size meaning that larger vesicles had a higher fluorescence intensity than smaller ones (data not shown). However, the data still showed that for vesicles incubated with 2C nanostructures the highest peak is associated with a complete NBD reduction which is in contrast to 1C nanostructure experiments where the highest peak is located at a much higher remaining fluorescence intensity. A small fraction of vesicles in the case of 1C nanostructures is completely reduced in their fluorescence which could be caused by dithionite leakage into the vesicles while they were moved upon dithionite addition. Fluorescence intensity traces show an exponential decay to almost 0 or to ~ 0.5 for vesicles incubated with 2C or 1C nanostructures, respectively (Supplementary Fig. 13b). Spikes in intensity are caused by vesicles temporarily being out of focus. The results are comparable to the assay performed in the main text. However, the slight improvements in the protocol effectively prevented vesicles from moving while still not exposing them to an osmotic shock which made results more consistent.

Supplementary Table 4. Alternative protocol of dithionite reduction assay (results shown in Supplementary Fig. 13).

Amount	Buffer conditions
20 µl	GUV solution in 100 mM sucrose, 20 mM HEPES pH7.4
24.5 µl	100 mM Glucose, 20 mM HEPES pH7.4
0.5 µl	0.5 % OPOE in TE20
5 µl	DNA nanostructures at 1 µM in TE20 buffer
	→ Incubation for approx. 150 min at room temperature<br>→ Record z-stacks of multiple areas containing vesicles
50 µl	10 mM dithionite in 80 mM glucose, 20 mM HEPES pH7.4
	→ Record z-stacks of multiple areas containing vesicles after one hour

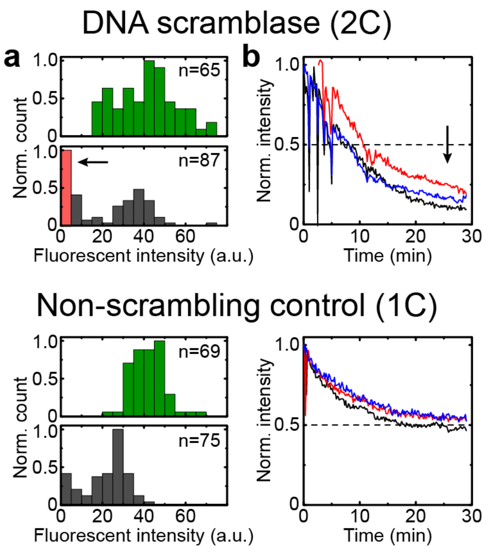

Supplementary Figure 13. Results of dithionite reduction experiments performed using an alternative measurement protocol. (**a**) Absolute NBD fluorescence intensities before (green) and approximately one hour after (grey) dithionite addition. Arrow and red column highlight a shift in the population down to no residual fluorescence intensity. Counts were normalized to the maximum for each histogram. The fluorescence intensity for 2C data has been scaled by the ratio of the average fluorescence intensity before dithionite addition of 2C-incubated vesicles over 1C-incubated vesicles. Number of analyzed vesicles is denoted by *n*. (**b**) Exponential reduction of NBD fluorescence from vesicles incubated with 2C and 1C nanostructures. The experiment was performed as described in Supplementary Table 4 but this time one field of view containing vesicles was imaged every 5 s over time. Fluorescence reduction below 0.5 indicates lipid scrambling. Large intensity spikes are caused by vesicles temporarily not being in focus. The shifted red trace in the top graph is caused by the vesicle entering the field of view only some moments after dithionite addition.

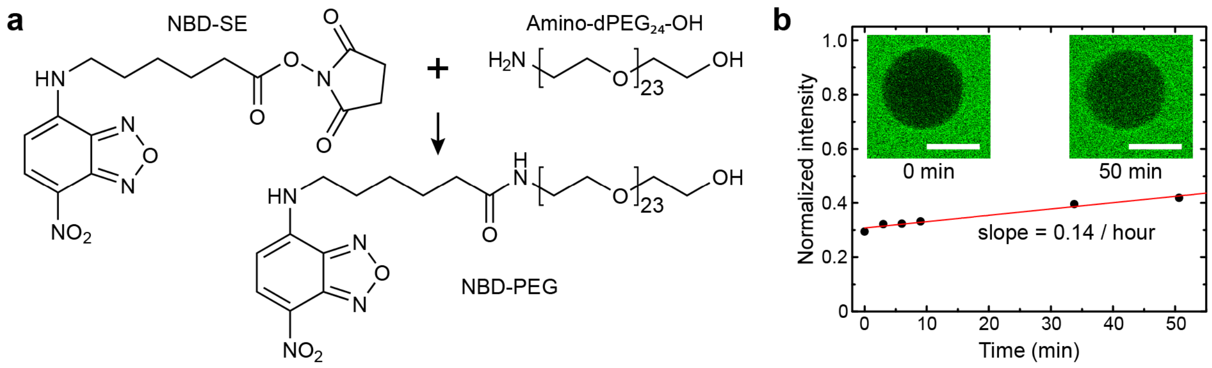

Supplementary Figure 14. Synthesis and membrane permeability of NBD-PEG. (**a**) Chemical structures of succinimidyl 6-(N-(7-nitrobenz-2-oxa-1,3-diazol-4-yl)amino)hexanoate (NBD-SE; Invitrogen), an NHS-ester of the fluorescent dye NBD, an amine-terminated 24-unit polyethylene glycol (PEG24; Quanta Biodesign), and their reaction product NBD-PEG. Dried NBD-SE and PEG24 were each diluted in 1:1 chloroform:methanol to final concentrations of 10 mg ml^{-1} (≈ 25 mM) and 137 mg ml^{-1} (≈ 128.3 mM), respectively. For the reaction, 100 µl NBD-SE and 20 µl PEG24 were mixed with 380 µl 1:1 chloroform:methanol and incubated for one hour at room temperature under constant movement. The solution was then blow-dried with nitrogen, desiccated for 10 minutes under vacuum and subsequently dissolved in 500 µl of 20 HEPES (pH 7.4) for a final concentration of ≈ 5 mM NBD-PEG. (**b**) POPC vesicles were prepared as described in Methods except that the dried lipids were hydrated in sucrose buffer containing 70 µM NBD-PEG. Vesicles were diluted in glucose buffer as used for dithionite reduction assays but supplemented with 70 µM NBD-PEG and incubated for two hours with 2C DNA nanostructures. Insets confocal microscopy images showing the fluorescence intensity directly after NBD fluorophores inside a vesicle were photobleached (0 minutes) and 50 minutes later (scale bars represent 5 µm). The graph displays the recovery of the fluorescence intensity inside the vesicle over time after photobleaching normalized to the intensity outside the vesicle at 0 minutes. Red line represents a linear fit. Its slope of 14 % per hour indicates a slight rate of NBD-PEG permeation back into the vesicle. Analogous experiments performed with 2-(N-(7-nitrobenz-2-oxa-1,3-diazol-4-yl)amino)-2-deoxyglucose (2-NDB-glucose; Invitrogen) yielded a full recovery of the fluorescence intensity inside the vesicle at time scales ≈ 10 minutes (data not shown) demonstrating comparatively rapid membrane permeation.

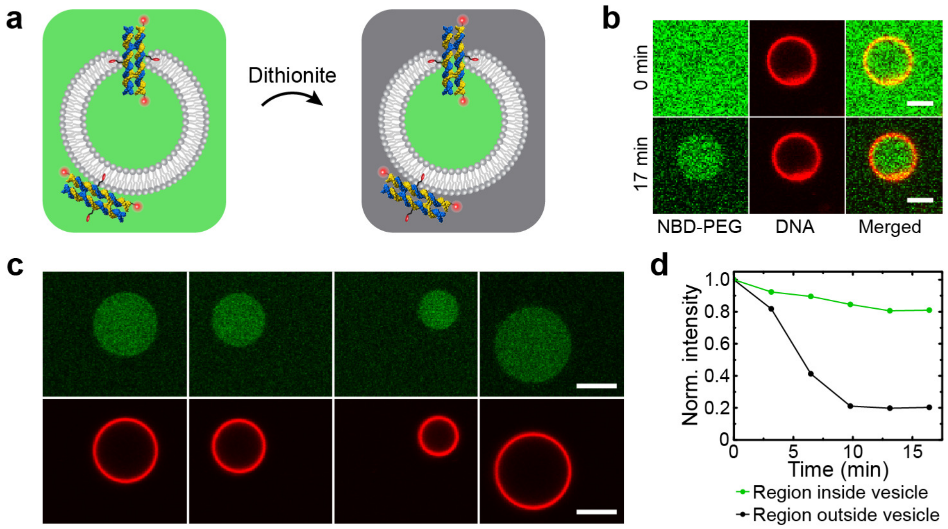

Supplementary Figure 15. Dithionite reduction assay performed on GUVs with equal concentrations of NBD-PEG inside and outside vesicles and incubated with 2C DNA nanostructures. POPC vesicles were prepared as outlined in Supplementary Fig. 14B. Dithionite reduction assay was performed analogously as described in Methods but with a final concentration of 70 µM NBD-PEG also present outside the vesicles. DNA nanostructures were continuously imaged every 10 s whereas NBD-PEG was imaged intermittently every 200 s (= every 20th frame). (**a**) Scheme illustrating how addition of dithionite would only reduce the fluorescence of NBD-PEG outside vesicles if 2C DNA nanostructures do not facilitate permeation of dithionite into the vesicles. (**b**) Confocal fluorescence microscopy images of GUVs incubated with the Cy3-labeled 2C DNA scramblase design (red) and with NBD-PEG (green) in solution at equal concentrations inside and outside the vesicles. The same vesicle is shown before and 17 minutes after dithionite addition. The third column displays a merged image of the red and green channels. Scale bars are 5 µm and the same for all images. (**c**) Vesicles at other locations within the chamber used in the assay described in **b** but imaged at time points between 37 and 48 minutes after dithionite addition. Scale bars are 5 µm and the same for all images. (**d**) Graph showing normalized NBD-PEG fluorescence intensity traces after dithionite addition from regions inside (green) and outside (black) the vesicle displayed in **b**. The intensity reduction for a region outside the vesicle follows similar kinetics as observed for the dithionite reduction in the scrambling assay (main text Fig. 4c). The NBD fluorescence intensity inside the vesicle, however, decreases at a much slower rate.

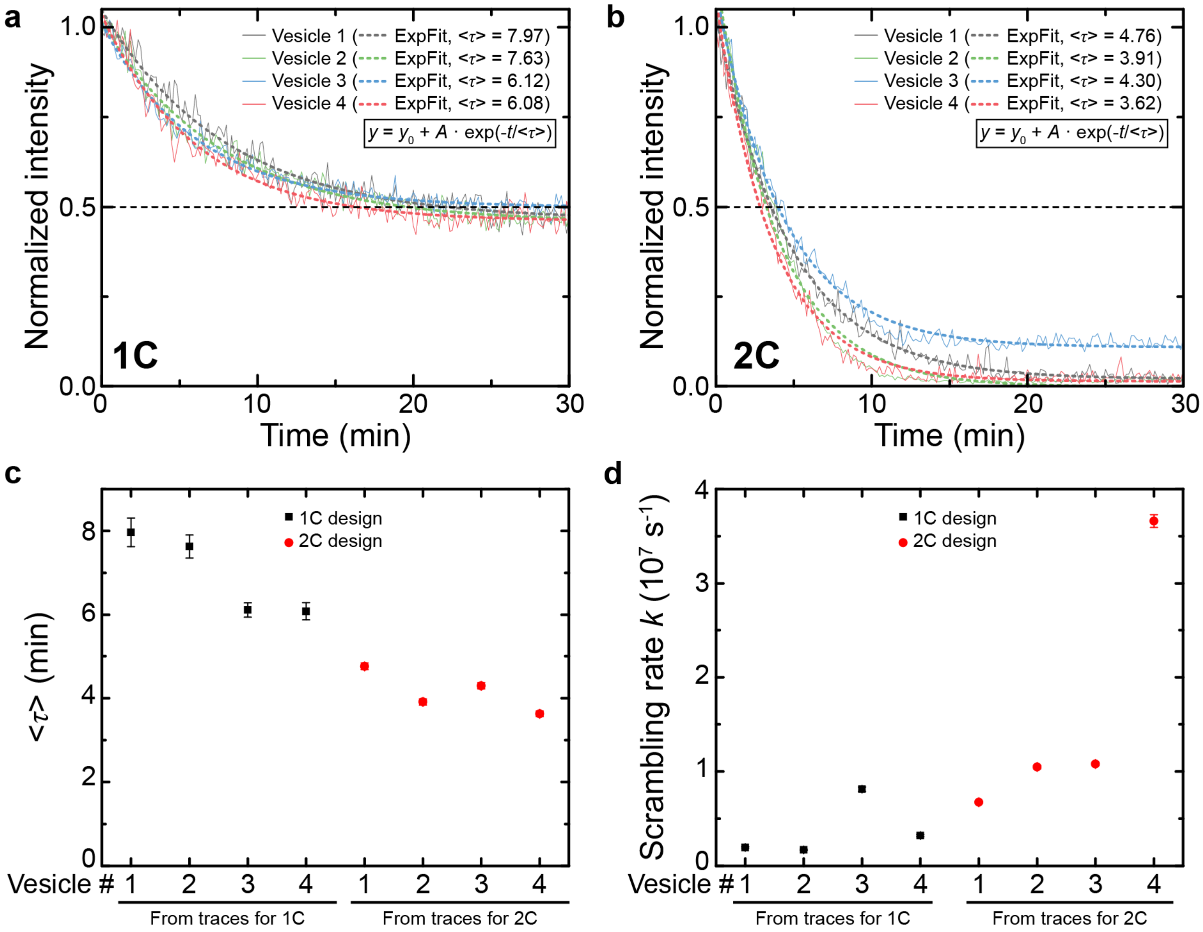

Supplementary Figure 16. Dithionite reduction rates determined from GUV experiments. (**a**,**b**) Exponential fits (dotted lines) of dithionite reduction traces (solid lines) shown in main text Fig. 4c for 1C and 2C nanostructures, respectively. Fit values $\langle\tau\rangle$ of decay rates are shown in the legend with the fitting equation below. (**c**) Graph comparing the characteristic decay times $\langle\tau\rangle$ (error bars denote fitting error) of the different dithionite reduction traces for 1C and 2C designs. (**d**) Scrambling rates approximated from characteristic decay times shown in **c** with error bars originating from error of $\langle\tau\rangle$. Using literature values for POPC lipids for Luzzati thickness $D_B = 3.68$ nm and area of a lipid $A = 0.683$ nm^2, the volume of a lipid can be determined[24]. By measuring the diameter of the specific vesicle, the total volume of the membrane can be calculated and divided through the volume of a single lipid to obtain the total number of lipids. For scrambling rate calculations, the number of lipids per vesicle without an additional factor was divided by $\langle\tau\rangle$ since simulations showed that the scrambling rate does not depend on the ratio of labeled lipids (main text Fig. 3d).

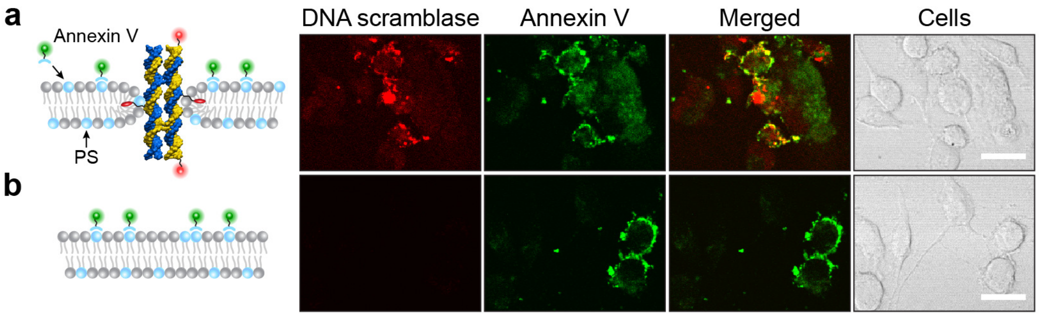

Supplementary Figure 17. Schematic illustrations and confocal microscopy images of scrambling experiments in human cells. (**a**) Same experiment and results as shown in main text Fig. 4g,h but kept for direct comparison to positive control. (**b**) Positive control experiment in which cells were incubated in the presence of 10 µM staurosporine (S5921, Sigma) following the same protocol as for DNA scramblase experiments. No signal was detected in the red channel illustrating sufficient spectral separation of the FITC dye from the Cy3. All imaging settings were the same for the equivalent images in all experiments. Scale bars are 20 µm.

Supplementary Note 5. Scrambling rate calculations regarding lipid diffusion.

The mean time $\langle\tau\rangle$ for a particle that diffuses on a sphere until it encounters and gets irreversibly captured by a single, immobile trapping region can be approximated by

$$\langle\tau\rangle \approx \frac{b^2}{2D}\left[ln\frac{b}{s} - \frac{1}{2}\right]$$

where s is the radius of the trapping region, b the diameter of the sphere and D the diffusion coefficient of the particle diffusing along the spherical surface (adapted from equation (6.2)[25] using $b = 2R$ and $d \approx s$ for the limit of a point-like diffusing particle and direct absorption upon encounter). This can be applied to estimating the mean time it takes for a phospholipid, that diffuses within the membrane of a vesicle with the diameter b, to be flipped (= captured) by our DNA-induced toroidal pore of radius s. The equation holds if $1 \gg s/b$ which is true for the size of our DNA nanostructure embedded in a giant unilamellar vesicle. With this equation, $\langle\tau\rangle$ was calculated for the average diameter of the vesicles used for the determination of scrambling rates (see Supplementary Fig. 16b) and compared to the experimentally determined values (see Supplementary Table 5).

Supplementary Table 5. Calculated and experimentally determined rates. N_{total} refers to the total number of lipids in a vesicle of diameter b. k is the lipid scrambling rate per scramblase.

s (nm)	D[26] ($\mu m^2\ s^{-1}$)	b (μm)	N_{total} (10^9)	$\langle\tau\rangle_{calc}$ (min)	$\langle\tau\rangle_{exp}$ (min)	k_{calc} ($10^7\ s^{-1}$)	k_{exp} ($10^7\ s^{-1}$)
2.3	7	19.4	3.47	3.61	4.76	1.60	1.62

Using $\langle\tau\rangle$, the scrambling rate k for a single DNA nanostructure can be estimated analogously as performed for experimental rates (see Supplementary Fig. 16) following

$$k = \frac{N_{total}}{\langle\tau\rangle}$$

With this equation, scrambling rates k were calculated and compared to the experimental values in Supplementary Table 5.

Supplementary References

1. Yoo, J. & Aksimentiev, A. In situ structure and dynamics of DNA origami determined through molecular dynamics simulations. *Proc. Natl. Acad. Sci. USA* **110,** 20099–20104 (2013).
2. Vanommeslaeghe, K. & MacKerell, A. D., Jr. Automation of the CHARMM General Force Field (CGenFF) I: bond perception and atom typing. *J. Chem. Inf. Model.* **52,** 3144–3154 (2012).
3. Maffeo, C., Yoo, J. & Aksimentiev, A. De novo reconstruction of DNA origami structures through atomistic molecular dynamics simulation. *Nucleic Acids Res.* **44,** 3013–3019 (2016).
4. Yoo, J. & Aksimentiev, A. Improved Parametrization of Li+, Na+, K+, and Mg2+ Ions for All-Atom Molecular Dynamics Simulations of Nucleic Acid Systems. *J. Phys. Chem. Lett.* **3,** 45–50 (2012).
5. Martyna, G. J., Tobias, D. J. & Klein, M. L. Constant pressure molecular dynamics algorithms. *J. Chem. Phys.* **101,** 4177–4189 (1994).
6. Feller, S. E., Zhang, Y., Pastor, R. W. & Brooks, B. R. Constant pressure molecular dynamics simulation: The Langevin piston method. *J. Chem. Phys.* **103,** 4613–4621 (1995).
7. Brünger, A. T. *X-PLOR, Version 3.1, A System for X-ray Crystallography and NMR*. (Yale University Press, 1992).
8. Phillips, J. C. *et al.* Scalable molecular dynamics with NAMD. *J. Comput. Chem.* **26,** 1781–1802 (2005).
9. MacKerell, A. D. *et al.* All-atom empirical potential for molecular modeling and dynamics studies of proteins. *J. Phys. Chem. B* **102,** 3586–3616 (1998).
10. Yoo, J. & Aksimentiev, A. Improved Parameterization of Amine–Carboxylate and Amine–Phosphate Interactions for Molecular Dynamics Simulations Using the CHARMM and AMBER Force Fields. *J. Chem. Theory Comput.* **12,** 430–443 (2016).
11. Miyamoto, S. & Kollman, P. A. Settle: An analytical version of the SHAKE and RATTLE algorithm for rigid water models. *J. Comput. Chem.* **13,** 952–962 (1992).
12. Andersen, H. C. Rattle: A 'velocity' version of the shake algorithm for molecular dynamics calculations. *J. Comput. Phys.* **52,** 24–34 (1983).
13. Batcho, P. F., Case, D. A. & Schlick, T. Optimized particle-mesh Ewald/multiple-time step integration for molecular dynamics simulations. *J. Chem. Phys.* **115,** 4003–4018 (2001).
14. Skeel, R. D., Hardy, D. J. & Phillips, J. C. Correcting mesh-based force calculations to conserve both energy and momentum in molecular dynamics simulations. *J. Comput. Phys.* **225,** 1–5 (2007).
15. Shaw, D. E. *et al.* Anton 2: Raising the Bar for Performance and Programmability in a Special-Purpose Molecular Dynamics Supercomputer. in *SC14: International Conference for High Performance Computing, Networking, Storage and Analysis* 41–53 (IEEE Press, 2014).
16. Nosé, S. A unified formulation of the constant temperature molecular dynamics methods. *J. Chem. Phys.* **81,** 511–519 (1984).

17. Hoover, W. G. Canonical dynamics: Equilibrium phase-space distributions. *Phys. Rev. A* **31,** 1695–1697 (1985).

18. Comer, J. & Aksimentiev, A. Predicting the DNA sequence dependence of nanopore ion current using atomic-resolution Brownian dynamics. *J. Phys. Chem. C* **116,** 3376–3393 (2012).

19. Siu, S. W. I., Vácha, R., Jungwirth, P. & Böckmann, R. A. Biomolecular simulations of membranes: Physical properties from different force fields. *J. Chem. Phys.* **128,** 125103 (2008).

20. Venkatesan, B. M. *et al.* Lipid bilayer coated Al2O3 nanopore sensors: towards a hybrid biological solid-state nanopore. *Biomed. Microdevices* **13,** 671–682 (2011).

21. Böckmann, R. A., Hac, A., Heimburg, T. & Grubmüller, H. Effect of Sodium Chloride on a Lipid Bilayer. *Biophys. J.* **85,** 1647–1655 (2003).

22. Li, C.-Y. *et al.* Ionic conductivity, structural deformation, and programmable anisotropy of DNA origami in electric field. *ACS Nano* **9,** 1420–1433 (2015).

23. Yoo, J. & Aksimentiev, A. Molecular Dynamics of Membrane-Spanning DNA Channels: Conductance Mechanism, Electro-Osmotic Transport, and Mechanical Gating. *J. Phys. Chem. Lett.* **6,** 4680–4687 (2015).

24. Kucerka, N., Tristram-Nagle, S. & Nagle, J. F. Structure of fully hydrated fluid phase lipid bilayers with monounsaturated chains. *J. Membr. Biol.* **208,** 193–202 (2005).

25. Sano, H. & Tachiya, M. Theory of diffusion-controlled reactions on spherical surfaces and its application to reactions on micellar surfaces. *J. Chem. Phys.* **75,** 2870–2878 (1981).

26. Macháň, R. & Hof, M. Lipid diffusion in planar membranes investigated by fluorescence correlation spectroscopy. *Biochimica et Biophysica Acta (BBA) - Biomembranes* **1798,** 1377–1391 (2010).
