# Supplementary material for: A synthetic enzyme built from DNA flips 107 lipids per second in biological membranes
Source: Nat Commun. 2018 Jun 21;9:2426. doi: 10.1038/s41467-018-04821-5 (PMC6013447; doi:10.1038/s41467-018-04821-5)
Supplement: Supplementary file 3 — Description of Additional Supplementary Files [file 41467_2018_4821_MOESM3_ESM.pdf]

Description of Additional Supplementary Files

File Name: Supplementary Movie 1
Description: All-atom simulation of lipid scrambling produced by a DNA nanostructure. The movie illustrates a 2.2 µs MD trajectory of a DNA nanostructure (blue and yellow) embedded in a DPhPE lipid membrane via cholesterol tags (semi-transparent red). The phosphorus atoms of the DPhPE lipid membrane are shown as light-yellow spheres. Except for one lipid (number 51, also highlighted in Fig. 2c of the main text), all other atoms of the lipid membrane are not shown, for clarity. Lipid number 51 is colored according to the atom type (C: cyan; O: red; N: blue; P: light yellow; H: not shown). The 1 M KCl electrolyte solution is not shown.

File Name: Supplementary Movie 2
Description: Additional example of spontaneous inter-leaflet transfer events occurring during the 2.2 µs all-atom MD simulations of the DPhPE system containing a DNA nanostructure. Supplementary Movie 2 features lipid #166, see Supplementary Fig. 3 for quantitative characterization of the transfer process.

File Name: Supplementary Movie 3
Description: Additional example of spontaneous inter-leaflet transfer events occurring during the 2.2 µs all-atom MD simulations of the DPhPE system containing a DNA nanostructure. Supplementary Movie 3 features lipid #203, see Supplementary Fig. 3 for quantitative characterization of the transfer process.

File Name: Supplementary Movie 4
Description: Additional example of spontaneous inter-leaflet transfer events occurring during the 2.2 µs all-atom MD simulations of the DPhPE system containing a DNA nanostructure. Supplementary Movie 4 features lipid #278, see Supplementary Fig. 3 for quantitative characterization of the transfer process.

File Name: Supplementary Movie 5
Description: BD simulation of lipid scrambling by a toroidal nanopore. The movie shows a collection of still microscopic configurations illustrating the 200 µs simulation trajectory of the $L = 24$ nm system. Both cut-away and top views of the same simulation system are shown in the top and bottom panels, respectively. The red-white-blue background in both panels represents the positiondependent potential applied in the simulation to account for the effect of the toroidal pore. The color map at the top panel shows the $Y - Z$ cross section of the system at $X = 0$ nm; the one at the bottom is the $X - Y$ cross section at $Z = 2$ nm. The color scale of the potential is shown at the right panel. The green dashed circle in the bottom panel indicates the location of the pore. The yellow and black spheres depict the fluorescent and the non-fluorescent lipids. Initially (at $t = 0$), the fluorescence of all lipids in the lower leaflets ($Z < 0$ nm) of the bilayer is reduced. During the simulation, lipids from the upper leaflet ($Z > 0$ nm) diffuse to the lower leaflet where they are reduced in their fluorescence (yellow beads turn black). The “Ratio” variable shows the ratio of the beads remaining in the upper leaflet that have never ventured to the lower leaflet from the beginning of the simulation to the total number of beads in the upper leaflet.
